# Ectopic Mediastinal and Lumbar Thyroid Tissue

**DOI:** 10.7759/cureus.18598

**Published:** 2021-10-08

**Authors:** Salman Khan, Madeeha Subhan Waleed, Deepak Verma, Mansoor Rahman

**Affiliations:** 1 Gastroenterology and Hepatology, Beth Israel Deaconess Medical Center, Harvard Medical School, Boston, USA; 2 Internal Medicine, Capital Hospital, Islamabad, PAK; 3 Internal Medicine/Family Medicine, California Institute of Behavioral Neurosciences & Psychology, Fairfield, USA; 4 Internal Medicine, Lady Reading Hospital MTI, Peshawar, PAK

**Keywords:** thyroid cancer, lumbar, mediastinum, thyroglobulin, ectopic thyroid tissue, : thyroid nodule

## Abstract

The thyroid gland is found in the neck and corresponds to the 2-4 tracheal cartilages. It is rarely found in other sites and is termed ectopic thyroid tissue (ETT) once found. In this report, we present a case of a 19-year-old female who was diagnosed with ETT in the lumbar region and posterior mediastinal region after total thyroidectomy for a large dominant thyroid nodule. ETT has a unique presentation and physicians should be vigilant to diagnose it correctly and promptly to decrease morbidity associated with the disease.

## Introduction

The thyroid gland is found in the neck and corresponds to the 2-4 tracheal cartilages. It is rarely found in other sites and is termed ectopic thyroid tissue (ETT) if found. The incidence of the localization of ETT in the posterior mediastinum has not been reported. The ETT is usually benign but is at risk of malignant transformation and can cause bleeding and compression of adjacent organs when it enlarges [1,2]. Surgical resection of the ETT may successfully treat the condition [1,3]. The most common location of ETT is the wall of the thyroglossal duct cyst but it can be found in other locations such as the ovaries, adrenal gland, and lungs [4-7]. The differential diagnosis of a mass found in the posterior mediastinum or lumbar region is fairly important and it is crucial to exclude other pathologies such as lymphomas, metastatic lesions from a malignant thyroid nodule, and dermoid cysts. All of these conditions require separate management methods. In this report, we discuss a rare case of mediastinal and lumbar ETT presenting post thyroid lobectomy.

## Case presentation

The patient was a 19-year-old female who presented with a thyroid nodule in the neck that had been discovered by her mother after she had returned from school. The patient had no neck pain, hoarseness of voice, or difficulty in swallowing. She had no history of exposure to any form of radiation either. The review of systems was normal. She was a non-smoker and a nondrinker. At the time of presentation, her vital signs were stable. Physical examination revealed no specific findings except for a palpable left thyroid mass. Chvostek’s sign was negative. Her thyroid function tests (TFTs) in March 2020 are shown in Table 1.

Thyroid ultrasound showed a complex nodule measuring 3.3 x 3.7 x 3.6 cm in the left thyroid lobe; no micro-calcification was seen and the right lobe of the thyroid was normal. Fine needle aspiration of the nodule showed a benign large complex nodule. Anti-thyroid antibodies were negative. Left thyroid lobectomy was performed and the pathology report showed an adenomatoid nodule. During the next visit, her physical examination revealed a lumbar mass, which had been previously considered a lipoma. The surgeon resected the lesion as he had a suspicion that it was not lipoma. Pathology reports came back as atypical thyroid tissue. Metastatic thyroid cancer was considered despite the atypical site of the metastasis. She ended up having a completion thyroidectomy. She had remnant ablation with radioactive iodine (radioiodine) I-131 after thyroidectomy. The post-ablative scan showed uptake in the neck and larger uptake in the chest. No uptake was seen in the lumbar area.

The MRI scan showed T2 and T1 isointense lesions in the right anterior paraspinal region, just to the right of the azygos vein at T9-T10 level. It measured 1.1 x 0.7 cm and showed mild enhancement. There was also central hypo enhancement. No additional enhancing lesions in the mediastinum were found. Post thyroidectomy, she was put on levothyroxine (LT4) 150 mcg for replacement and thyroid-stimulating hormone (TSH) suppression. Her laboratory results in May 2020 are shown in Table 1. Repeat thyroid uptake showed diminished uptake in the mediastinal mass. The dose of levothyroxine was titrated to keep TSH low around 0.2 mIU/L. Thyroglobulin was persistently high. She returned after two months in July 2020 and her laboratory reports at that time are shown in Table 1.

She was referred to a tertiary care center for a second opinion. The repeat MRI chest showed a decrease in the size of the mediastinal mass. The neck lymph nodes (II and IV) had reactive lymphadenopathy. The case and pathology slides were reviewed by many pathologists and they concluded that this was an anomalous variation of the thyroid gland and not a malignancy, as no clear-cut malignant cells could be seen on the histopathology slides and they just showed colloid filled follicles. It was labeled as Ectopic Thyroid Tissue by Endocrine and Tumor Boards in Chicago. The dose of levothyroxine was suggested to be kept high enough for TSH suppression. Her repeated TFTs in January 2021 are shown in Table 1. Her thyroglobulin levels have trended down, and she has been followed up regularly at our outpatient clinic. Her most recent TFTs in August 2021 are shown in Table 1.

**Table 1:** Thyroid function tests TSH: thyroid-stimulating hormone; FT3: free triiodothyronine; FT4: free thyroxine; ATA: anti-thyroid antibodies

Month	TSH (mIU/mL)	FT3 (pg/mL)	FT4 (ng/dL)	ATA
March 2020	0.878	2.5	1.0	Negative
May 2020	0.185	2.5	1.3	Negative
July 2020	0.005	2.7	1.6	Negative
January 2021	0.0025	3.0	1.7	Negative
August 2021	0.153	3.0	1.3	Negative

## Discussion

The thyroid gland develops from the first and second pharyngeal arch through endodermal protrusion in the third to seventh week of gestation; it descends anteriorly from the foramen caecum forming a thyroglossal duct. The abnormal migration of thyroid tissue during the development leads to the formation of ETT [3,5]. Ectopic thyroid is reported to be prevalent in one in every 10,000-300,000 people in the general population and one in every 4,000-8000 among patients with thyroid disease [8]. ETT exhibits morphological variation and dysplasia is the predominant form of variation [9]. ETT mostly has an asymptomatic presentation. Our patient was asymptomatic at the time of presentation as well. However, based on the size and location of the tissue, it can present with various symptoms like hoarseness of voice, dysphagia, or foreign body sensation when present in or around the neck region [5]. Our patient had a mass in the lumbar region and a thyroid nodule and no additional complaints. The histopathologic examination of the ectopic mass showed atypical thyroid tissue.

A similar case of ectopic lumbar thyroid tissue was reported by Lin et al. in 2021 in a 67-year-old woman who presented with left lumbago and leg pain [9]. Thyroid scintigraphy, ultrasonography, CT scan, and histological examination of the mass are used in the diagnosis of ectopic thyroid mass [8]. Posterior mediastinal ETT is a rare occurrence and has been reported to comprise 1% of all mediastinal mass [1]. An abnormal thyroid uptake scan of the patient revealed the ectopic lesion in the posterior mediastinum. The malignant transformation of ectopic mass is rare. Our patient had a benign ETT in the mediastinal region, which was confirmed on histopathology. Masses should be surgically removed due to the increased risk of malignant transformation, progressive enlargement, and compression of adjacent structures in the mediastinum [1]. Surgical resection is the mainstay of therapy followed by radioiodine ablation [8]. Surgical resection is achieved through thoracotomy as it provides complete resection and better visualization and has relatively lower mortality and morbidity rates [1]. Our patient was treated surgically and underwent radioiodine therapy - RAI ablation resulting in the shrinkage of the mediastinal mass. She is being followed up regularly with thyroglobulin levels testing and imaging.

## Conclusions

Despite the rarity of ETT, it should be considered as a possibility while dealing with a mediastinal mass. Differentiating it from other mediastinal masses is of utmost importance as it can impact the management and outcome. Histopathological specimens should be obtained to differentiate between malignancy and ETT. Suspected ETT should undergo both functional and anatomic characterizations through a CT scan to help in the medical treatment of hypothyroidism and hyperthyroidism associated with ETT. ETT has a unique presentation and physicians should be vigilant to diagnose it correctly as misdiagnosis can lead to delays in treatment and consequent complications.
